# New insight of high-intensity interval training on physiological adaptation with brain functions

**DOI:** 10.20463/jenb.2018.0017

**Published:** 2018-09-30

**Authors:** Min Chul Lee, Sung Ki Lee, Suk Yool Jung, Hyung Hoon Moon

**Affiliations:** 1 Department of Sports Medicine, CHA University, Pocheon Republic of Korea

**Keywords:** high-intensity interval training (HIIT), resistant wheel running (RWR), brain functions, hippocampus, brain-derived neurotrophic factor (BDNF)

## Abstract

**[Purpose]:**

Exercise effectively enhances physiological adaptation, performance, and health-related markers in healthy individuals and diseased populations. However, the type and amount of optimal exercise remain controversial. High-intensity interval training (HIIT) took the top spot in the American College of Sports Medicine worldwide survey on fitness trends in 2018.

**[Methods]:**

We review information on the effect of HIIT on physiological adaptation and the novel role on brain functions.

**[Results]:**

HIIT is a more cost-effective way to improve physiological adaptations with aerobic capacity, and it also enhances brain functions such as hippocampus-dependent learning and memory.

**[Conclusion]:**

In this review, we provide insight on the utility of HIIT in improving performance and highlight suggestions for future research.

## INTRODUCTION

It is well known that physical activity and exercise training is effective ways to enhance physiological adaptations including those of the cardiovascular and musculoskeletal system, as well as brain function. Studies have shown that exercise improves various markers of cardiovascular and skeletal muscle adaptation^1-3^. It also increases the central nervous system such as adult hippocampal neurogenesis (AHN)^4,5^, which is associated with hippocampus-dependent spatial learning and memory function^6,7^. This evidence suggests that exercise may enhance synaptic transmission and plasticity. Although previous studies have demonstrated that exercise is linked to central and peripheral changes, the optimal type and amount to generate these beneficial effects remain controversial.

Data from the American College of Sports Medicine (ACSM) demonstrate that high-intensity interval training (HIIT) is a consistently high-ranking trend on the forecast in 2018^8^. In the past 12 years, ACSM’s Health & Fitness Journal (FIT) has circulated electronic survey among fitness professionals around the world to determine health and fitness trends. The survey lists possible trends identified by a comprehensive group of professionals from five sectors of the industry: corporate, clinical, community, commercial, and academia. HIIT entered the number one spot in 2018 and has been within the top 3 positions for 3 years. HIIT is considered the most popular exercise and this is indeed a trending exercise. It is popular in gyms across the world despite warnings from industry experts that it can have a relatively high risk and can cause injuries.

Here we review some of the mechanisms responsible for improved skeletal muscle metabolic control and changes in cardiovascular function in response to HIIT, as well as the potential health-related implications for subjects. Moreover, we are speculating on the effects of practical application of HIIT on performance. Although, the underlying mechanisms may be different in less-trained subjects, responses in athletes may help to determine why HIIT is such a potent exercise stimulus for brain function.

### Characteristic of HIIT and physiological adaptations

Although it has been among the top fitness trends, many of the ACSM surveys comments claim that clients liked the program for a short time, and then looked for another exercise. Others warned that despite its popularity, there was a potentially high rate of injury. Others working with clinical populations in medical fitness centers said they would like to try it with their patients but would substitute high-intensity with moderate-intensity interval training.

HIIT workouts are designed to have bursts of maximum-effort, typically approximately 20 to 90 seconds, followed by a short period of rest or low-intensity recovery. These exercise programs are usually performed for less than 30 minutes, but some last much longer. The goal is to recover enough so that the trainee can exert maximum effort again in the next work interval. Therefore, HIIT exercise can vary in terms of the length of low-intensity recovery, but not the length of high-intensity

HIIT is infinitely variable with the specific physiological adaptations induced by this form of training determined by a numerous of factors including the precise nature of the exercise stimulus, including intensity, duration and number of intervals performed, as well as the duration and activity patterns during recovery. It has been shown to affect aerobic capacity, mitochondrial fatty acid oxidation, and cardiovascular risk as effectively as endurance training (ET)^9-11^. Compared with a basis or when estimated energy expenditure is equivalent, HIIT is an effective alternative to traditional endurance training, inducing similar or even superior changes in a range of physiological adaptation, performance, and health-related markers in healthy individuals and diseased populations^12,13^.

Little is known regarding the effects of HIIT, but growing evidence suggests that it stimulates physiological remodeling similar to moderate-intensity continuous training, despite a substantially lower time commitment and reduced total exercise volume^14^. The most common model employed in low-volume HIIT studies has been the Wingate test, which consists of a 30 s “all out” cycling effort against a supra-maximal workload. Subjects typically perform four to six work bouts separated by approximately 4 min of recovery, for a total of 2–3 min of intense exercise during a training session that lasts approximately 20 min. As little as six sessions of this type of training, totaling approximately 15 min of all-out cycling exercise over 2 weeks, increased skeletal muscle oxidative capacity as reflected by the maximal activity and/or protein content of mitochondrial enzymes^15^. It has also directly compared 6 weeks of Wingate-based HIIT has been shown to be equivalent to traditional endurance training (Table 1).

**Table 1.** Summary of study protocols in HIIT. Six weeks of high-intensity interval training (HIIT) versus traditional endurance training. $\dot{V}$_O_2peak__, peak oxygen uptake. From Burgomaster *et al*. (2008).

Variable	HIT group	Endurance group
Protocol	30s x 4-6 repeats, 4.5 min rest(3 sessions per week)	40-60 min cycling (5 sessions per week)
Training intensity (workload)	'All out' maximal effort (~500 W)	65% of $\dot{V}$_O_2peak__ (~150W)
Weekly training time commitment	~10 min (~1.5 h including rest)	~4.5 h
Weekly training volume	~225 kJ	~1150 kJ

From Burgomaster et al. (2008). $\dot{V}$_O_2peak__, peak oxygen uptake.

### Potential underlying molecular mechanisms

The molecular mechanisms underlying skeletal muscle metabolic adaptations to HIIT have recently been investigated. Given the potency of HIIT to increase mitochondrial capacity, it is perhaps not surprising that researchers have examined the influence of HIIT on the activation of peroxisome-proliferator-activated receptor γ co-activator (PGC)-1α^16^. A significant breakthrough in unraveling the cellular events that promote mitochondrial biogenesis was the discovery of PGC-1α, an inducible coactivator that regulates the coordinated expression of mitochondrial proteins encoded in the nuclear and mitochondrial genomes^17^.

In the skeletal muscle, PGC-1α has emerged as a key regulator of mitochondrial biogenesis that responds to neuromuscular input and prevailing contractile activity. A single bout of endurance exercise induces a rapid and sustained increase in the PGC-1α gene and protein in the skeletal muscles^18^, whereas, muscle-specific overexpression of PGC-1α results in a large increase in functional mitochondria^17^, improvements in whole-body VO_2max_, a shift from carbohydrate to fat fuels during submaximal exercise, and improved endurance performance^19^. AMPK and p38 MAPK are two important signaling cascades that converge on the regulation of PGC-1α and consequently the regulation of mitochondrial biogenesis^20^. AMPK and p38 MAPK phosphorylate and activate PGC-1α expression, a known regulator of PGC-1α. This, in turn, increases PGC-1α protein by binding to and activating the CREB site on the PGC-1α promoter^21^.

**Fig. 1.**
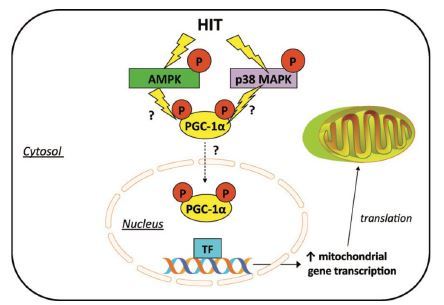
Potential intracellular signaling mechanisms involved in HIIT-induced mitochondrial biogenesis. HIIT has been shown to activate AMPK and MAPK. From Gibala et al. (2012).

Evidence suggests that exercise intensity is a key factor influencing PGC-1α activation in human skeletal muscle. Loss of function studies has challenged the absolute requirement of PGC-1α during training-induced changes in muscle mitochondrial biogenesis, angiogenesis, and fiber type changes^22^. Recent observations place PGC-1α as a central player in orchestrating many of the oxidative adaptations to exercise. In this respect, acute HIIT increases PGC-1α mRNA post-exercise^23^. Similar to endurance exercise, acute HIIT may activate PGC-1α by increasing its nuclear translocation^24^. The increase in nuclear PGC-1α following low-volume HIIT coincides with increased mRNA expression of several mitochondrial genes, suggesting that a program of mitochondrial adaptation is engaged with these short bursts of intensity exercise (Fig. 1)^25^.

### Possible role in brain plasticity

New light has recently been shed on the large number of effects of HIIT. It is considered to be a time-efficient alternative to traditional endurance training that elicits similar metabolic responses in humans^15,23^ and animals ^10,11^. The effects of HIIT on the cognitive functions and its related underlying mechanisms have yet to be investigated. Here, the choice of the HIIT was based on our earlier findings which possibility of voluntary resistance wheel running (RWR) to improve the brain functions. To address this, here we aimed to establish a model of HIIT for animals and examine whether HIIT effectively enhances hippocampal-related spatial learning and memory, based on the possible mechanisms underlying hippocampal BDNF signaling.

Although, acute and chronic voluntary resistance wheel running increased high-energy expenditure with load, but did not increase running distance (half of control), it also replicated the effects of traditional wheel running on learning and memory associated with brain-derived neurotrophic factor (BDNF) signaling and induced hippocampal adaptations such as neurogenesis in rats^26,27^. Additionally, it provides an inventory of newly changed gene expression and identify alterations in a number of transcriptional pathways in RWR rat hippocampal plasticity^28^.

RWR allows for a given load on a running wheel and is a useful exercise model to increase work levels as energy expenditure without physical and psychological stressors. Enhanced activities of oxidative enzyme citrate synthase together with fast-twitch plantaris muscle hypertrophy is possible because of the relatively high intensity and short duration of intermittent exercise^29,30^. This supports previous reports demonstrating that HIIT has more pronounced effects on the fast-twitch muscle fiber^15^. Thus, it is possible the HIIT is a distinct type of high-intensity exercise because it adapts to high-energy demands by increasing the strength, which is clearly different than the traditional ET model. Also, we could believe that present HIIT manner in rats reproduced in the adaptations close to the studies conducted in humans.

It is reasonable to assume that BDNF is a key protein supporting the growth, development, and survival of neurons. Physical activity increases hippocampal BDNF mRNA and protein^31^, which, in turn, promotes hippocampal-dependent cognitive function and neurogenesis^32,33^. Physical exercise increases hippocampal BDNF mRNA and protein, which, in turn, promotes adult neurogenesis^7^. Changes in BDNF signaling is apparently necessary for exercise to impact hippocampal plasticity in rodents; blocking BDNF signaling prevents exercise-induced learning and memory^7^ and neurogenesis^34^. These results, which support previous studies, suggest that HIIT represents an effective model to enhance spatial cognitive functions with increased hippocampal BDNF signaling, even with lower exercise volume and time.

RWR data demonstrated that a lower volume and shorter time of exercise similar to HIIT is more effective to enhance hippocampus-dependent learning and memory associated with hippocampal BDNF signaling than endurance training. These findings suggest that HIIT is an effective alternative to traditional endurance training, producing similar or even greater changes in a range of physiological, performance, and health-related markers. HIIT may be similar to RWR, intermittent exercise, and produces shorter distances but higher work levels, it also an efficient exercise that plays a beneficial role in brain functions. Altogether, intermittent exercise of high-intensity, such as HIIT, improves muscle adaptation and aerobic capacity, while also potentially improving brain function.

## CONCLUSION

Altogether, it helps the health and fitness industry make critical activity programming and business decisions. The results of this annual survey may help the health and fitness industry make some very important investment decisions for future growth and development. In this review, we discuss some of the mechanisms responsible for improving physiological responses and changes in cardiovascular function in response to HIIT as well as the potential health-related implications for athletes and patients with chronic diseases. Furthermore, we speculate on the practical applications of HIIT in performance then important from a public health promotion, given that lack of time remains one of the most commonly cited barriers to regular exercise. Although the underlying mechanisms of HIIT may differ from those seen in less-trained subjects, responses in elite athletes may help our understanding of why HIIT is such a potent exercise stimulus. Moreover, recent studies have shed light on HIIT increases the role of training method in preventing and/or treating brain functions. Future studies are needed to uncover the role of HIIT parameters on brain function.
